# Facilitating Change in School Health: A Qualitative Study of Schools’ Experiences Using the *School Health Index*

**Published:** 2006-03-15

**Authors:** S Bryn Austin, Teresa Fung, Adena Cohen-Bearak, Kacey Wardle, Lilian WY Cheung

**Affiliations:** Division of Adolescent Medicine, Children’s Hospital; Dr Austin is also affiliated with the Harvard Prevention Research Center and the Department of Society, Human Development, and Health, Harvard School of Public Health, Boston, Mass; Department of Nutrition, Simmons College, and Department of Nutrition, Harvard School of Public Health; Harvard Prevention Research Center, Department of Society, Human Development, and Health, Harvard School of Public Health; Department of Nutrition, Harvard School of Public Health; Harvard Prevention Research Center and Department of Nutrition, Harvard School of Public Health, Boston, Mass

## Abstract

**Introduction:**

As school-based efforts increase to address the epidemic of childhood obesity, a priority for health professionals and educators will be to identify effective tools appropriate for use in schools to help guide health promotion programs and policies. This article describes the results of a qualitative research study examining school staff and community members' experiences working with the Centers for Disease Control and Prevention's *School Health Index*, a self-assessment and planning tool that addresses nutrition and physical activity.

**Methods:**

In-depth interviews were carried out with faculty, staff, and community collaborators in nine public schools that were using the *School Health Index* to develop nutrition and physical activity initiatives for students. Interviews were conducted twice: once after a school had completed the *School Health Index* and once approximately 1 year later. Transcript data from interviews with 34 participants were analyzed using thematic analysis.

**Results:**

Findings indicated that school experiences differed markedly depending on whether they received help from an outside facilitator to work with the *School Health Index*. Unlike staff in schools working on their own, school staff working with outside facilitators described completing the *School Health Index* in a collaborative way, creating action plans, and working as a team to implement health promotion initiatives. In addition, the involvement of an outside facilitator supported schools in undertaking more complex tasks with a greater degree of collaboration across the school and local communities in order to achieve goals.

**Conclusion:**

Outside facilitators may significantly enhance schools' efforts to work with the *School Health Index* and influence the organizational strategies they use to implement health promotion initiatives.

## Introduction

Most of the nation's public schools have a variety of nutrition and physical activity programs of some type in place, yet multiple countervailing pressures act on schools to impede these efforts to improve nutrition and physical activity opportunities for students (1-5). Recognizing the potential for schools to provide more healthful nutrition and physical activity environments for the nation's young people and to address the child and adolescent obesity epidemic (6,7), the Centers for Disease Control and Prevention (CDC) introduced the *School Health Index: A Self-Assessment and Planning Guide* (SHI) in 2000 (8) to provide educators with a tool to guide and inform school health promotion initiatives. The SHI is a self-assessment tool for primary and secondary schools designed to help school staff evaluate the strengths and weaknesses of school-based health promotion programs and policies and plan for further improvement. Structured around CDC's coordinated school health program model, SHI assessment items were developed from empirical research and policies and practices recommended in two CDC reports: *Guidelines for School Health Programs to Promote Lifelong Healthy Eating* and *Guidelines for School and Community Programs to Promote Lifelong Physical Activity Among Young People* (9,10).

The original version of the SHI focused on nutrition and physical activity, and subsequent versions have been expanded to also address tobacco use and injury prevention. The SHI manual, which is provided to schools for free by CDC, is approximately 100 pages and includes eight modules for assessment of different program areas of the school. Instructions encourage a team approach to completing the self-assessments with input from people throughout the school and local communities. Schools are advised to identify an internal school SHI coordinator and a school team to complete some or all of the modules, score the results of the modules, develop and prioritize recommendations based on the results for health promotion initiatives, and carry out work to implement these health promotion initiatives.

The SHI has been available for several years, yet little is known about the strategies that schools use to conduct self-assessments and to carry out the day-to-day work required to implement initiatives for program and policy changes. We conducted a qualitative study to describe school health promotion teams' experiences working with the SHI and their subsequent efforts to address nutrition and physical activity in the year following the completion of the assessment.

## Methods

### Study sample and research design

In summer 2001, study recruitment was begun to identify all eligible schools in New England. Schools were considered eligible if they were public primary or secondary schools or both and were planning to begin using the SHI for the first time within 6 months. Recruitment efforts included sending out notices to state departments of education and departments of public health in New England, posting notices on an Internet listserv for school health professionals, and meeting with professional contacts within the state departments of education and departments of public health in the region. Six public schools from three states in New England were identified that met eligibility criteria. All of these schools agreed to participate. In addition, three public schools from one school district in the Midwest were identified and enrolled in the study. Schools in New England, located in urban and suburban areas, included preschool, elementary school, and middle school grades. The Midwestern district, located in a rural area, included elementary, middle, and high school grades. 

In each school, the staff person responsible for coordinating the team's work with the SHI also served as the study liaison, helping us identify members of the school community working with the SHI, whom we then invited to participate in two research interviews. The semistructured individual interviews were conducted twice: approximately 1 month after each school completed the SHI self-assessment (Time 1) and then again approximately 1 year later (Time 2). Interviews lasted an average of 45 minutes and were audiotaped and transcribed. Informed consent was obtained from participants before interviews. Participants were offered an incentive of $25 value. The Harvard School of Public Health institutional review board approved this study.

Two semistructured interview guides were developed for the study. Interview questions at Time 1 elicited information about how the decision was made to work with the SHI, experience with the SHI manual, strategies used to complete the assessment, the role of the SHI coordinator and, if applicable, the role of a facilitator from outside the school, the types of health-promoting initiatives identified by the group as priorities, and the organizational strategies used to implement these initiatives. Questions at Time 2 explored the ways that members of the team worked together during the school year subsequent to completing the SHI self-assessment, the roles of the SHI coordinator and outside facilitator, the types of initiatives pursued since Time 1, and the organizational strategies used to carry out these initiatives.

### Data analysis

Research staff analyzed transcripts of interviews using the thematic analysis technique (11,12) in which an iterative process was used to identify central concepts, categories, and themes that arose during interviews. Through a series of analysis meetings, the research team developed a taxonomy of analysis codes based on emerging findings (11,12). Transcripts were then analyzed by two independent coders (A.C. and K.W.) using NVivo software (QSR International, Doncaster, Victoria, Australia). Preliminary analyses indicated that participating schools differed in the degree of facilitation assistance they received to work with the SHI. In addition, facilitation assistance appeared to relate in important ways to the schools' description of the strategies they used to complete the SHI self-assessment and to carry out work on initiatives. To examine these patterns in more depth, we grouped data from schools into three categories based on the degree of facilitation assistance they received: low facilitation (LF), moderate facilitation (MF), and high facilitation (HF). In the LF group, which consisted of four schools, staff in each school completed the SHI with no outside assistance. In the MF group, which consisted of three schools working together in a single team, a district administrator helped staff to complete the SHI but did not continue to meet with them subsequently. In the HF group, which consisted of two schools, an outside facilitator from a community-based nonprofit organization worked with each school to complete the SHI and continued to meet regularly with them throughout the school year. All transcripts were analyzed by comparing the three facilitation categories.

## Results

In total, 52 interviews were conducted with 34 participants (nine men, 25 women). Twenty-four interviews were with the LF group, 11 with the MF group, and 17 with the HF group. Approximately half of the interviews were conducted at Time 1 and half at Time 2. Study participants included school district staff, community agency staff, principals, school nurses, cafeteria managers, physical education (PE) teachers, classroom teachers, and one student. Schools participating in the study were ethnically and economically diverse. In three of the LF schools, approximately 85% of students were eligible for free or reduced-price lunches, and in the fourth school, 25% were eligible; in the MF schools, approximately 40% of students were eligible for free or reduced-price lunches; and in both HF schools, approximately 85% of students were eligible for free or reduced-price lunches.

### Time 1 results

#### Team structure and process for completing the SHI instrument

There were important differences by facilitation level in how well school teams followed CDC instructions for completing the SHI. In the LF schools, staff tended not to follow the instructions provided in the SHI manual. One school in the LF group distributed the SHI to staff and had them fill it out individually like a survey rather than complete it as a team. In another school, the SHI coordinator discussed the SHI with staff members individually, tabulated results, and came up with an average score. When asked about the SHI team structure, one participant from an LF school said, "Well, quite frankly, until today, I didn't quite realize we were part of the team." In contrast, MF and HF schools more consistently followed the instructions and worked as a team to complete the SHI, involving people from across the school community and, to some degree, the local community. They discussed the SHI during group meetings, then followed up with additional meetings to develop goals and action plans. In the MF and HF groups, decisions were made through consensus on scoring the SHI assessment and on goals and action plans.

#### Roles of facilitator

The MF group completed all of the SHI modules in one long meeting led by an outside facilitator, and then the team continued to meet monthly without the facilitator to discuss the team's action plan. Two staff members took on the role of team leaders and coordinated the monthly meetings and activities. The outside facilitator for the MF group, who worked on the district level, did not attend monthly team meetings but did provide guidance and technical assistance to the team as it worked on its action items. The two schools in the HF group completed three modules of the SHI at a series of monthly meetings led by the outside facilitator, and then the teams continued to meet monthly with the facilitator to work on their action plans.

Members of the MF and HF groups perceived the facilitator as having a motivating presence in meetings, describing that they felt the facilitator kept the team focused, helped it achieve consensus on decisions, and guided it in following through with identified action items. A staff member in the HF group commented on how the team's experience might have been different had they not had a facilitator: "I think it probably would have been much more frustrating to try to get some policies or just get some consensus. I think it would have been more difficult. She [the facilitator] kind of keeps us on track."

#### Administrative support

Participants from all three groups perceived that support from the school administration was essential. They described the following ways that they felt school administrators could support the efforts of health promotion teams: 1) ensure that staff have protected time to meet as a team; 2) participate in the team's meetings; and 3) authorize implementation of action plans. Before starting the SHI self-assessment, teams in both the MF and HF groups garnered support from the school administrators. In the MF group, the principal of the middle school participated in the team meetings. In the HF group, before initiating work on the SHI, the facilitator met with the school principals and staff and obtained a letter of support from the superintendent and agreement from the principals to allow the team to implement its recommendations. The facilitator from the HF group commented, "You absolutely need the approval, the blessing, of the principal to make this work." In three of the four LF schools, full support from the school principal was not obtained before embarking on the SHI self-assessment; the principals did not participate in team meetings nor did they agree to implement recommendations from the team.

### Time 2 results

At Time 2, participants in all three groups spoke positively about the SHI and felt they became more aware of the importance of nutrition and physical activity while also noting that pressure from standardized testing and limited resources and staff time were obstacles to their efforts. The three groups diverged, however, during the school year (after completing the SHI) in the way that they described organizational strategies they used and the types of initiatives they pursued.

#### Team process from Time 1 to Time 2

During the follow-up year, the three groups differed in their reliance on teams and teamwork. In two LF schools, staff occasionally met in pairs or small groups to discuss the SHI results and the schools' health promotion needs, but none of the four LF schools held regular team meetings. In addition, LF schools did not develop and document action plans or work as a team toward defined goals for health promotion. 

In the MF group, participants met monthly without the facilitator, created an action plan, and worked toward goals. Two staff members continued in the role of team leader, and the outside facilitator provided guidance and technical assistance as needed. In the HF schools, the teams continued to meet monthly with the outside facilitator. Using what they described as consensus decision making, they identified goals and an action plan, focusing on a few priority issues, and worked as a team toward implementing those changes. A participant from a community agency in the HF group commented:

I think the *School Health Index* is a tool that's appropriate for a school that has a team in place, ready to begin and do some formal assessment and develop a formal action plan to improve the nutrition and physical activity environment in the school. I think that if you walk into any school cold, . . . you're going to meet with some resistance.

#### Action plans and follow-through

The kinds of activities and goals emerging from schools in each of the facilitation levels appeared to differ in two potentially important ways: task complexity and degree of collaboration used to achieve goals. Participants in LF schools described a variety of changes achieved by Time 2, most of which involved effort by one or two people. Changes mentioned included assigning a substitute teacher to teach PE classes that the regular PE teacher could not fit into her schedule, ordering exercise videotapes for PE classes and classroom resources on nutrition and physical activity, and allowing students access to the gym before and after school. With her school district facing drastic budget cuts, one PE teacher in the LF group considered the fact that PE classes had not been completely eliminated from the curriculum to be a positive result of their having completed the SHI assessment, which she believed had sensitized administrators to the need for PE. She also intimated that she doubted policy improvement in PE could be expected in her school:

There was more of a push for more activity. We need more. [But] the schedule in and of itself cannot be changed. . . . Only the eighth graders get physical education two times a week. Everybody else gets it once a week. That's how the schedule allows it.

In the MF group, activities achieved by the team in the year following completion of the SHI required substantial changes in school food service operations, including reducing the frequency that french fries were served, rearranging the lunch line so students passed by vegetables first, installing milk machines, and substituting baked for fried foods. In addition, the MF group planned and implemented a student health fair and staff wellness day and established a subsidized health club membership for staff. 

By the time of the second wave of interviews, however, several changes instituted by the school district, including the departure of a key advocate in the administration of the SHI team's work, had recently occurred. Members of the MF group felt these changes led the team to become less active. A staff member from the MF group explained:

I think I would call this [second] year more maintenance. We have not stopped doing what we've been doing. We just haven't done probably as much. Maybe the momentum has slowed slightly. We still feel very strongly about it. We still would like to make changes. . . .

In the HF group, the two schools' teams shared the same outside facilitator and began working on initiatives in similar ways. However, in one of the HF schools, near the end of the year, the district slated the school for closure, so the team stopped working on the planned changes. The other HF school differed from all the other schools in the study in that its team decided to focus on a single health issue that team members considered both important for student health and realistic for the team to address within the school year, given limited resources and time. In this school, the team chose to promote student hand washing before meals and snacks. Ensuring that students can wash their hands before meals and snacks is included as a recommendation in the school environment module of the SHI. Coordinating hand washing by hundreds of students every day required significant planning and collaboration across different sectors of the school community. The team planned this new initiative, gained approval of the school superintendent, developed an evaluation plan, acquired and installed hand-sanitizer dispensers, trained teachers on the new policy, and implemented the policy schoolwide to ensure that students washed their hands before eating snacks and meals. A participant from the school observed, "The fact that the team worked as a team and included everybody else, I think really helped move this thing along quickly."

Participants in all three groups described insufficient funding for staff, sale of nonnutritious snack food to fund operating costs for school food services, and lack of physical activity space and equipment as barriers to health-promoting change. School staff from all three groups also reported that health programs and policies were at times seen by administrators as conflicting with student academic achievement. One teacher from an LF school described state and federal programs that she felt presented obstacles to health promotion efforts: "Well, unfortunately . . . right now their priority is the [state standardized testing] and the No Child Will Be Left Behind. . . and unfortunately, maybe as a result, other issues are left behind."

#### Roles of facilitator

Participants in the MF and HF groups described three central roles played by outside facilitators:

**1. Administration buy-in:** Facilitators helped garner support from the administration before the SHI assessment was begun. They secured commitment from the administration to allow the team to meet regularly and to implement initiatives decided upon by the team.

**2. Team structure: **Facilitators guided schools toward using a team structure, as recommended by CDC, to complete the SHI assessment and implement initiatives. Facilitators supported team structure by ensuring that 1) key stakeholders from across the school community and, to some degree, the local community were included on the team; 2) team meetings were held regularly for continued contact and communication among team members; and 3) team meetings were facilitated with an emphasis on consensus building and management of group tensions and conflict.

**3. Team sustainability:** Facilitators helped teams become sustainable by maintaining team motivation and team cohesion through coaching and focus on feasible tasks to foster a sense of concrete gains and accomplishments.

One participant from a community agency in the HF group emphasized the importance of the facilitator:

Everybody in the school has a job to do, and this is in addition to their job. And having a facilitator who can lead — well, not necessarily lead, but help them lead, that can spot some of the stumbling blocks and help them get over those stumbling blocks faster, quicker, more efficiently and move towards completing action steps — is invaluable to the success of this project, to implementing the *School Health Index*.

She saw the SHI as an important tool but not sufficient to effect change in schools: "A tool alone is not effective," she explained. "It's got to go in there with an advocate, with somebody to help it, help implement that tool."

## Discussion

Across the nation, public schools are grappling with budget cuts, increasing demands on teachers' time, and political pressures to improve student performance on standardized tests. The schools in our study were no different. Despite the economic and academic pressures faced by all the schools in our study, school staff members working with the SHI with the help of outside facilitators, unlike those carrying out the work on their own, described completing SHI assessments in a collaborative way, creating action plans that involved complex tasks and required collaboration across multiple arenas of the school, and working consistently as a team during the school year to implement health promotion initiatives. The involvement of an outside facilitator appeared to be linked with meaningful differences in the strategies used by teams to work with the SHI and to implement initiatives.

Staten et al recently reported results of a study of the SHI implemented in 13 public schools in Arizona, where the vast majority of students were Latino and from low-income households (13). Similar to our findings, Staten et al conclude that outside facilitators offered support and guidance to schools carrying out SHI assessments and developing health promotion goals. Also consistent with our results, they found that limited staff time and resources were important barriers to action (13).

Seeking to go beyond simply documenting differences across facilitation levels, we extended our analyses to include patterns and relationships in the data that may inform hypotheses about the role of facilitators in shaping schools' organizational strategies in carrying out health promotion initiatives. We observed that the process that schools used to carry out the SHI self-assessment appeared to be closely linked to their subsequent reports of sustained team meetings throughout the school year, development of action plans, task complexity, degree of collaboration across the school community, and follow-through on concrete tasks to implement initiatives.

Our findings suggest that the process that schools use to complete the SHI self-assessment is not incidental. Rather, we propose that use of a team structure and team decision making with a facilitator should be viewed as a part of the larger organizational strategy that may engender commitment to action (14) needed to sustain the day-to-day work required to implement initiatives. In addition, we propose that group decision making and the locus of decision making (14,15) are informative ways to characterize schools' organizational strategies to work with the SHI and plan and implement health promotion initiatives. Furthermore, we propose that these characteristics of organizational strategies may be integral to understanding the role of outside facilitators in school-based health promotion initiatives.

Based on our findings, we have developed a preliminary hypothetical model of the potential impact of outside facilitators on school-based health promotion efforts. Within this model, shown in the Figure, outside facilitators may be predicted to positively affect health promotion initiatives through their roles related to administration buy-in, team structure, and team sustainability. Perhaps by shifting to some degree the locus of decision making from administrators to health promotion teams, as we observed in MF and HF schools in our study, and supporting the creation and maintenance of teams with the capacity to make group decisions, facilitators may help to engender commitment to action. Our model suggests that the emergence of these decision-making capacities in the teams may be predicted to then positively affect implementation of initiatives in terms of 1) development of action plans, including strategies for continuing to build political support for initiatives within the school community; 2) task complexity; and 3) degree of collaboration across the school community. Although our findings are suggestive, our model is preliminary, and continued research is needed to further characterize facilitator roles and organizational strategies used in schools working on health promotion initiatives.

**Figure:**
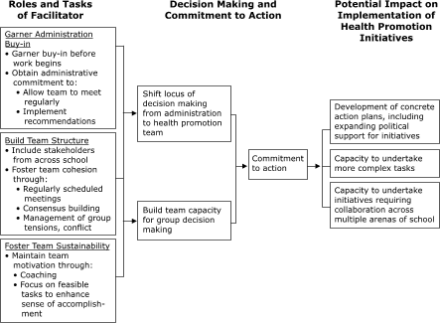
Inductively derived hypothetical model of a facilitator's role in supporting a school's work with CDC's *School Health Index *and implementation of health promotion initiatives.

There were several limitations to our study. The majority of the schools were in New England, with just three in the Midwest; schools in other regions may have different experiences. In addition, our study relied on self-reported data and did not include a supplemental objective data source, which may have led us to overlook some important aspects of the schools' experiences.

Based on our findings, we can make several recommendations for schools planning to use the SHI as a tool to guide their health promotion work. Before beginning the SHI self-assessment, it is important for schools to garner support from school administrators, perhaps even in writing. This buy-in should include a commitment to allow protected time for the team to complete the SHI and work on initiatives and commitment to implement team recommendations. Working with an already-established team may enhance this work. The team may benefit from including representatives from several different arenas of the school and local communities. A facilitator from outside the school, perhaps from the school district or a community agency, who has a commitment to work with the team for an extended period may be important, perhaps even essential, for some schools. Together, the facilitator and team need to anticipate and plan for likely obstacles.

The federal Child Nutrition and WIC Reauthorization Act of 2004 included a new requirement that all school districts participating in the U.S. Department of Agriculture subsidized meals program must establish a school wellness policy for improving student nutrition and physical activity by the beginning of the 2006–2007 school year (16). This new federal policy is intended to prompt schools nationwide to develop comprehensive initiatives to promote healthful nutrition and physical activity programs and policies (17). The SHI could be used as a tool to introduce public health technical expertise to schools as they develop school wellness policies and programs, but our findings suggest this is not sufficient. Schools are complex organizations, and the processes of change may be enhanced by involvement of outside facilitators to support the efforts of health promotion teams. Use of the SHI as a self-assessment tool coupled with attention to strategies relating to team building and decision making may enhance efforts to build commitment to and capacity for action in schools. Ultimately, these strategies may foster meaningful improvement in school nutrition and physical activity environments for the nation's youth.
